# Transcriptomic data of bovine ovarian granulosa cells of control and High A4 cows

**DOI:** 10.1016/j.dib.2021.107217

**Published:** 2021-06-11

**Authors:** Alexandria P. Snider, Sarah M. Romereim, Renee M. McFee, Adam F. Summers, William E. Pohlmeier, Scott G. Kurz, John S. Davis, Jennifer R. Wood, Andrea S. Cupp

**Affiliations:** a Animal Science, University of Nebraska–Lincoln, P.O. Box 830908, C203 ANSC, Lincoln, NE 68583-0908, USA; b School of Veterinary Medicine and Biomedical Sciences, University of Nebraska-Lincoln, P.O. Box 830905, Lincoln, NE 68583-0905, USA; c Animal and Range Sciences, New Mexico State University, Knox Hall Room 202; MSC 3-I Las Cruces, NM 88003, USA; d Olson Center for Women's Health, University of Nebraska Medical Center, 983255 Nebraska Medical Center, Omaha, NE 68198-3255, USA; e VA Nebraska-Western Iowa Health Care System, Omaha, NE 68105, USA

**Keywords:** High androgen microenvironment, Granulosa cell microarray, Cell cycle arrest, microRNA, Bovine

## Abstract

Microarray analysis using Affymetrix Bovine GeneChip 1.0 ST Array to determine RNA expression analysis was performed on somatic granulosa cells from two different groups of cows classified based on androstenedione concentration within the follicular fluid (Control vs High A4) of estrogen-active dominant follicles. The normalized linear microarray data was deposited to the NCBI GEO repository (GSE97017 - RNA Expression Data from Bovine Ovarian Granulosa Cells from High or Low Androgen-Content Follicles). Subsequent ANOVA determined genes that were enriched (≥ 1.5 fold more) or decreased (≤ 1.5 fold less) in the High A4 granulosa cells compared to Control granulosa cells and analyzed filtered datasets of these differentially expressed genes are presented as tables. MicroRNAs that are differentially expressed in Control and High A4 granulosa cells are also reported in tables. The standard deviation of the analyzed array data in relation to the log of the expression values are shown as a figure. Ingenuity Pathway Analysis determined upstream regulators of differently expressed genes as presented in a table. These data have been further analyzed and interpreted in the companion article “A High-Androgen Microenvironment Inhibits Granulosa Cell Proliferation and Alters Cell Identity” (McFee et. al., 2021 [1].

## Specifications Table

**Table:** 

Subject	Biology
Specific subject area	Reproductive Physiology
Type of data	Table Figure
How data were acquired	RNA Microarray
Data format	Normalized Analyzed Filtered Raw data deposited in NCBI Repository GEO
Parameters for data collection	Isolation of estrogen active somatic granulosa cells from an environment with androstenedione (A4) concentrations of ≤ 20 ng/mL (Control) or ≥ 40mg/mL (High A4).
Description of data collection	Microarray analysis of bovine granulosa cells from an excess androgen environment
Data source location	Institution: University of Nebraska-Lincoln and University of Nebraska-Medical Center City/Town/Region: Lincoln and Omaha, NE Country: USA
Data accessibility	Repository name: NCBI Repository GEO Data identification number: GSE97017 Direct URL to data: https://www.ncbi.nlm.nih.gov/geo/query/acc.cgi?acc=GSE97017
Related research article	R.M. McFee, S.M. Romereim, A.P. Snider, A.F. Summers, W.E. Pohlmeier, S.G. Kurz, R.A. Cushman, J.S. Davis, J.R. Wood, A.S. Cupp, A High-Androgen Microenvironment Inhibits Granulosa Cell Proliferation and Alters Cell Identity, Mol. Cell Endocrinol. 111288 https://doi.org/10.1016/j.mce.2021.111288

## Value of the Data

• The follicular control granulosa cell transcriptome data can be compared to previously published bovine gene expression analyses [2,3], for corroboration.
• The list of identified genes that are differently expressed in a high androstenedione environment can inform future physiological research on the altered regulation of genes in follicular granulosa cell.
• The prediction of upstream regulators of differently expressed genes can inform future research on regulation of follicular granulosa cells that lead to the development of a high androstenedione (A4, High A4) environment.

## 1 Data Description

**Fig. 1:**
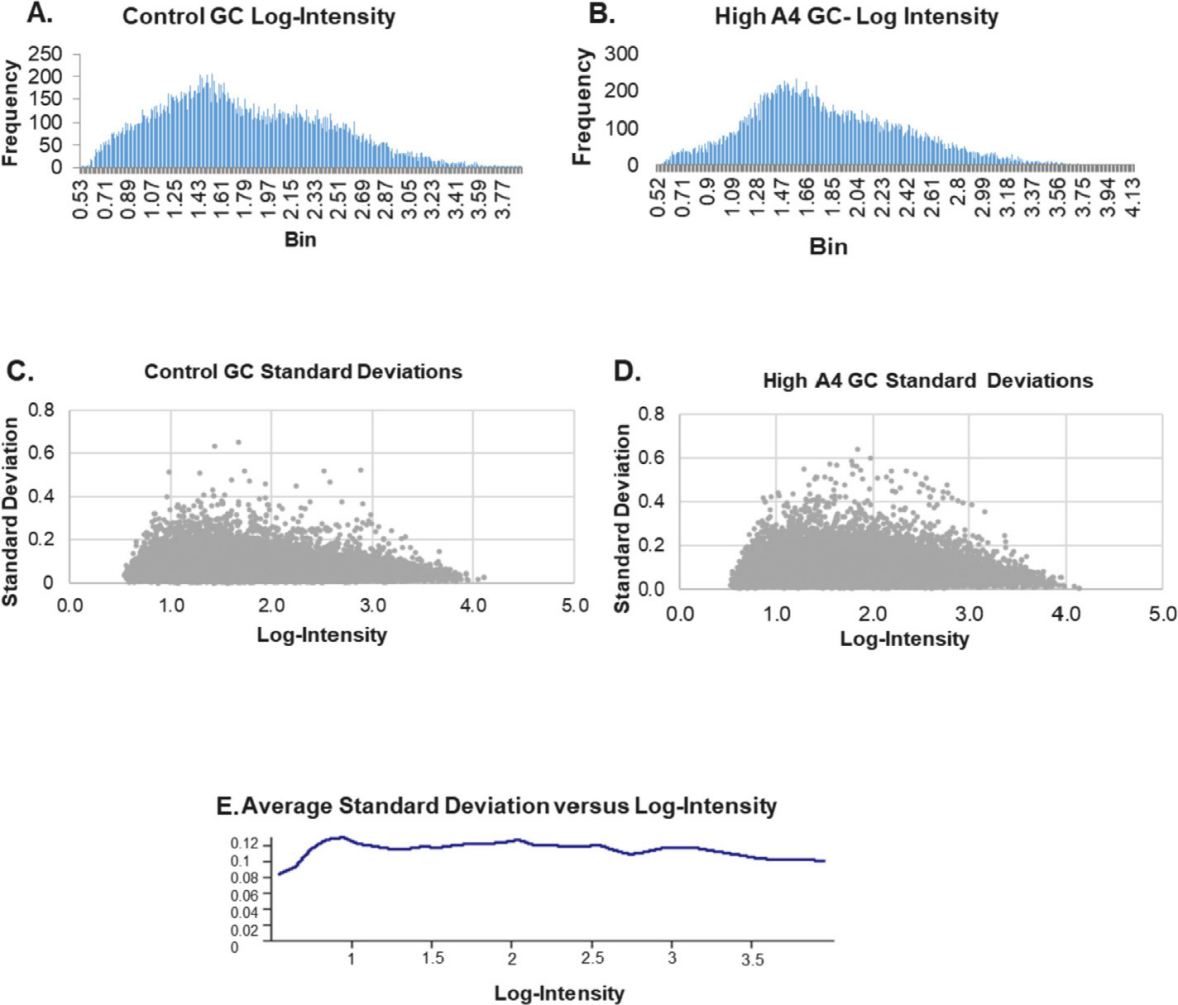
Microarray data log-intensities and standard deviations. A histogram of the frequency of microarray targets at a given average log-intensity for each granulosa cell group (A &B). The standard deviations versus each log-intensity are graphed as scatter plots for each granulosa cell group (C &D). The average standard deviation for each granulosa cell group and microarray replicates versus log-intensity is plotted in (E).

The microarray was analyzed to determine the noise frequency, log-intensity and standard deviation between the control and High A4 granulosa cells (Fig. 1).

Microarray analysis of granulosa cell mRNA from estrogen active dominant follicles from control and High A4 cows (n = 4/group) showed altered expression (*P* < 0.05 and Fold change > 1.5) of several genes involved with cell cycle regulation (Supplementary Table 1). The differently expressed genes were either enriched or depleted in the granulosa cell transcripts of High A4 cows and compared to other cell types located within the ovary (large luteal cell (LLC), small luteal cell (SLC) and theca cell (TC)) (Supplementary Table 2). Based on the IPA analysis, predicted upstream regulators (Supplementary Table 3) and predicted targets of miRNAs (Supplementary Table 4) were determined based on the differently expressed genes in the High A4 granulosa cells. The genes that are associated with follicular or luteal cells were shown to be altered in High A4 granulosa cells (*P* < 0.05 and Fold change > 1.5) (Supplementary Table 5).

## 2 Experimental Design, Materials and Methods

### 2.1 Study design

The University of Nebraska-Lincoln Institutional Animal Care and Use Committee approved all procedures and facilities used in these experiments. The University of Nebraska-Lincoln is fully AAALAC accredited and follows NIH guidelines. Cows (75% Red Angus, 25% MARC III) from the physiology herd located at the Eastern Nebraska Research and Extension Center (ENREC) were utilized for these experiments. Twenty-six cows over several reproductive cycles were classified into High A4 or Control cows. To do this, estrous cycles of cows were synchronized with prostaglandin F2alpha (PGF2α; 25 mg/mL; Lutalyse, Pfizer Animal Health) injected 14 days apart and dominant follicles (largest follicle) was aspirated 12–36 h after the second PGF2α injection. After follicular fluid estrogen and progesterone concentrations were measure via radioimmunoassay as described previously [4], to determine these follicles were estrogen active, androstenedione (A4) was measured in follicular fluid of estrogen active dominant follicles [6,7] and females were classified as Control or High A4 based on the dominant follicular fluid environment of A4. High A4 ≥40 ng/ml and Control A4≤20 ng/ml.

For the current experiment, estrous cycles of 10 High A4 and 10 Control cows were synchronized with a modified Co-Synch protocol using gonadotropin releasing hormone (GnRH, 100 mg; Cystorelin, Boehringer Ingelheim Animal Health) and a controlled internal drug release device (CIDR; 1.38 g progesterone, Zoetis) for 7 days with a PGF2α (25 mg/mL; Lutalyse, Pfizer Animal Health) injection at CIDR removal [4]. This protocol was utilized so all follicle waves that developed would be more synchronized and the dominant follicles would be at similar stages of development,

Ovariectomy was performed approximately 36 h after CIDR removal. Upon ovariectomy, the largest antral follicle from each cow's ovaries was aspirated/dissected and the granulosa cells [5] and follicular fluid were isolated as described previously [4,5]. Follicular fluid was analyzed for estrogen, progesterone and androstenedione [4,5]. Follicles with a follicular fluid E2:P4 ratio greater than 1 were classified as estrogen-active (EA; [6,7]).

### 2.2 RNA Isolation

Granulosa cells from four cows with follicular fluid (A4 concentration) at the highest range of the High A4 group (mean A4 = 356 ng/mL, SD = 148; range 203-597 ng/mL) and four cows with follicular fluid (A4 concentrations) at the lowest range of the Control group (mean A4 = 1.9 ng/mL, SD = 0.39; range 1.40-2.50 ng/mL) were utilized. The granulosa cells from the estrogen active dominant follicles from the 8 selected cows were then homogenized in Tri-reagent (Sigma-Aldrich) for RNA isolation as discussed previously [4,5].

### 2.3 Microarray analysis

For microarray analysis, 200 ng RNA for each sample (n = 4 animals for each group; High A4 and Control) were submitted to the University of Nebraska Medical Genomics Core facility where the Affymetrix Bovine GeneChip® Gene 1.0 ST Array RNA expression analysis was performed. Full transcriptome data is available from the NCBI GEO repository, Series GSE97017. The microarray results were normalized with Robust Multi-Array Averaging and this was conducted at the Genomics Core facility.

### 2.4 Data processing

Array analysis was performed with normalized microarray results using the National Institute of Aging array tool (NIA) (http://lgsun.grc.nia.nih.gov/ANOVA/) for Analysis of Variance (ANOVA) to determine hierarchical clustering, and correlation between replicates (Fig. 1). All bioinformatic analyses were performed on transcripts above a linear noise threshold of 100. Raw data counts for each cow and genes differentially expressed in the Control and High A4 granulosa cells are in Supplementary Table 1.

The NIA array data was inserted into the Ingenuity® Pathway Analysis (IPA; Winter 2016 release, Qiagen) program which identifies each annotated gene and predicts cell function outcomes. No canonical pathways were found to be significant with the 2016 release nor with an updated analysis (2021 January update, Qiagen).

Granulosa cell enriched or depleted transcripts were compared between High A4 and Control granulosa cells within IPA and then to theca (TC), small luteal cells (SLS) and large luteal cells (LLS) (Suplementary Table 2) via IPA comparison which have been described previously [5]. IPA analysis provided a list of predicted upstream regulators (Supplementary Table 3).

TargetScan Release 7.1 (June 2016) was utilized to determine potential miRNA targets for mammalian microRNAs (http://www.targetscan.org/vert_71/). For this program the miRNA sequence is inserted into the program and it shows all of the predicted genes that the particular miRNA targets. These targets are shown in Supplementary Table 4. Comparisons of Follicle or Luteal cell shared genes from previously published data [5] were compared and are shown in Supplementary Table 5.

## Ethics Statement

All animals were humanely treated and cared for in accordance with University of Nebraska-Lincoln IACUC guidelines. The University of Nebraska-Lincoln is fully AAALAC accredited and follows guidelines as followed by NIH guidelines. Transcriptomes of granulosa cells were evaluated in this study. Females were used in this study, as we are only interested in the mechanisms involved with granulosa cell function, which is only present in female mammalian species.

## CRediT Author Statement

**Alexandria P. Snider:** Data curation, Formal analysis, Investigation, Methodology, Project administration, Software, Validation, Visualization, Writing – original draft, Writing – review & editing; **Sarah M. Romereim:** Data curation, Formal analysis, Investigation, Methodology, Project administration, Software, Validation, Visualization, Writing – original draft; **Renee M. McFee:** Formal analysis, Investigation, Software, Validation, Visualization; **Adam F. Summers:** Data curation, Formal analysis, Investigation, Project administration; **William E. Pohlmeier:** Data curation, Formal analysis, Investigation; **Scott G. Kurz:** Data curation, Formal analysis, Investigation; **John S. Davis** Conceptualization, Funding acquisition, Investigation, Resources, Supervision, Writing – review & editing; **Jennifer R. Wood:** Conceptualization, Data curation, Funding acquisition, Investigation, Methodology, Resources, Supervision, Writing – review & editing; **Andrea S. Cupp:** Conceptualization, Data curation, Formal analysis, Funding acquisition, Investigation, Methodology, Project administration, Resources, Supervision, Writing – review & editing.

## Declaration of Competing Interest

The authors declare that they have no known competing financial interests or personal relationships which have or could be perceived to have influenced the work reported in this article.

## Appendix Supplementary materials

Image, application 1

Image, application 2

Image, application 3

Image, application 4

Image, application 5
